# Cancer awareness among adolescents in second-level education: a mixed methods systematic review

**DOI:** 10.1093/her/cyaf014

**Published:** 2025-05-12

**Authors:** Stephanie Lawrence, Serena FitzGerald, Josephine Hegarty, Mohamad M Saab

**Affiliations:** Catherine McAuley School of Nursing and Midwifery, University College Cork, Cork T12 AK54, Ireland; Catherine McAuley School of Nursing and Midwifery, University College Cork, Cork T12 AK54, Ireland; Catherine McAuley School of Nursing and Midwifery, University College Cork, Cork T12 AK54, Ireland; Catherine McAuley School of Nursing and Midwifery, University College Cork, Cork T12 AK54, Ireland

## Abstract

This systematic review aimed to synthesize evidence from studies that explored cancer awareness among adolescents. The systematic review was conducted in accordance with the Joanna Briggs Institute’s (JBI) guidelines. Seven electronic databases were systematically searched for studies published between January 2010 and March 2022. The search was updated in April 2024. Data extraction and quality appraisal were performed. Data were synthesized narratively. A total of 21 studies were included for review. Overall, adolescents were found to have low cancer symptom awareness and to report several perceived barriers to symptomatic presentation for cancer. Adolescents also seemed underinformed about modifiable lifestyle behaviours associated with cancer, which has the potential to lead to a higher level of engagement in health risk behaviours. Findings from this review indicate the importance of actively promoting cancer awareness among adolescents. This has the potential to lead to increased knowledge and help-seeking for symptoms of cancer among adolescents, which in turn would lead to earlier diagnosis and ultimately more positive patient outcomes.

## Introduction

Globally, 300 000 new cancer cases are diagnosed among adolescents each year [1–3]. Smoking, alcohol abuse, poor physical activity, obesity, poor nutrition, and exposure to the sun without adequate protection are key modifiable cancer risk factors among adolescents [4–7]. In the UK, around 2000 adolescents are diagnosed with cancer annually [8] and in the USA, about 5000 to 6000 adolescents aged 15 to 19 years are diagnosed with cancer each year [2, 3].

Early detection, appropriate and timely diagnosis and treatment are fundamental in reducing cancer morbidity and mortality among adolescents [9, 10]. Adolescents experience rare cancers, such as those of the testes, ovaries, and thyroid; however, leukaemia in adolescents constitutes the largest cancer burden globally, followed by brain and nervous system cancers [11].

Cancer in adolescents is different from cancer diagnosed in other age groups due to differences in cancer types, risk factors, tumour biology, prognosis, and survivorship [12]. In addition, compared with older patients with cancer, adolescents have a higher risk of long-term and late effects, including infertility, sexual dysfunction, cardiovascular disease, and future cancers [13–15].

Adolescence has been recommended as an ideal phase to promote health since, during this stage, most future health-related lifestyles, behaviours, habits, and attitudes are formed [5, 16–18].

Providing information about lifestyle cancer-related behaviours would potentially encourage adolescents to engage in healthier lifestyle choices that may persist into adulthood [5]. The implementation of effective interventional cancer awareness programmes could help reduce delays in diagnosis and improve cancer survival by increasing awareness of cancer symptoms and risk factors among adolescents [19].

The term ‘cancer awareness’ has been widely used in the adult population to explore knowledge of cancer symptoms, risk factors, self-examination, and screening [20–23].

Low cancer symptom awareness is associated with help-seeking delay [24–26]. Adolescents may delay help-seeking for symptoms of concern. This can negatively impact early diagnosis and treatment outcomes [15]. In a study by Niksic *et al*. [26], younger people reported lowest awareness scores for cancer symptoms and were less likely to recognise potential warning signs of cancer, such as unexplained lumps or swelling. According to a study by Al Qadire *et al*. late presentation for cancer is due to a lack of awareness of warning signs and risk factors of cancer [27]. Low cancer symptom awareness is influenced by factors such as ethnicity and gender, while perceived barriers to reporting symptoms include practical and service-related obstacles [28].

In recent years, there has been a strong focus on cancer awareness among adolescents [29–49], adolescents’ preferences for cancer education [43], the effectiveness of cancer educational programmes [50], and the effect of targeted education on adolescents’ knowledge of health risk behaviours pertaining to cancer [33, 34, 37, 40, 41, 44, 46, 47, 49]. Early interventions are encouraged to promote healthy behaviours from a young age [31, 33, 34, 37, 40, 41, 44, 46, 47, 49]. However, research has shown that adolescents are often unaware of and misinformed about cancer [31, 38]. In a study by Di Giuseppe *et al*. one third of adolescent participants were at high risk of developing cancer due to modifiable lifestyle habits associated with cancer including smoking, alcohol abuse, poor physical activity, and poor eating habits [6]. Smoking from a young age for example can significantly increase the risk of lung cancer [51].

Several learning theories indicate that comprehensive cancer prevention education, combined with positive modeling and personal engagement, can foster healthy behaviours that persist in adulthood. For example, the Social Learning Theory (Bandura, 1977) suggests that observing positive health behaviours in others and their benefits can motivate adoption of similar habits [52]. Behaviourism (Skinner, 1938) theorizes that understanding long-term consequences of risky behaviours can deter their practice [53]. Cognitivism (Piaget, 1936) emphasizes how adolescents can process and integrate cancer risk information with existing health knowledge, leading to informed decision-making [54]. Finally, constructivism (Vygotsky, 1978) proposes that adolescents can build personal understanding of healthy lifestyles through interactive educational experiences [55]. Therefore, it is important to ensure that adolescents are aware of health risk behaviours pertaining to cancer and are motivated to engage in preventative behaviours which help mitigate the risk of cancer later in life [30, 32, 34, 36, 44, 46, 47].

To the best of our knowledge a recent synthesis of evidence exploring cancer awareness, help-seeking, and health risk behaviours among adolescents has not been conducted. Thus, the aim of this systematic review was to synthesize current evidence on cancer awareness among adolescents. The objectives of this review include the exploration of adolescents’: (i) cancer awareness; (ii) knowledge of cancer signs and symptoms; (iii) knowledge of cancer risk factors; (iv) help-seeking for cancer symptoms; (v) barriers and facilitators to help-seeking for cancer symptoms; and (vi) awareness of and engagementin health risk behaviours linked to cancer.

## Methods

This mixed methods systematic review was conducted in accordance with the Joanna Briggs Institute’s (JBI) [56] methodology for mixed methods systematic reviews. The 27-item Preferred Reporting Items for Systematic Reviews (PRISMA) checklist guided the reporting of this review [57] and is presented in (Supplementary Information [SI] A).

### Eligibility criteria

The review inclusion and exclusion criteria were predetermined in accordance with the population, exposure, and outcomes (PEO) framework [58]. The included studies comprised findings relating to: adolescents aged 10 to 19 years as defined by the Sawyer *et al*. [17]; described or measured cancer awareness, knowledge of cancer signs and symptoms, knowledge of cancer risk factors, knowledge of health risk behaviours, and help-seeking in the context of cancer symptoms; conducted in secondary school settings as defined by The International Standard Classification of Education [59]; and conducted in countries ranking very high and high on the Human Development Index (HDI) [60]. Such countries were chosen as education systems and expected years of schooling differ among low and very low HDI countries. Qualitative and quantitative studies using descriptive designs were included. Pre-post studies were included, yet only pre-test findings were extracted in line with the review aim.

Studies were excluded if they were not related to cancer; focused on treatments for cancer or living with cancer; focused on specific cancer types or specific health risk behaviours; and/or involved adults. Moreover, studies not written in English, review papers, conference abstracts, theses and dissertations were excluded. Studies where post-test-data only were provided were also excluded [61–63].

### Search strategy

The following electronic databases were systematically searched to identify potentially relevant studies: CINAHL, ERIC, APA PsycINFO, APA PsycARTICLES, Psychology and Behavioural Sciences Collection, Education Full Text (H. W. Wilson), and MEDLINE. Search terms such as adolescen*, ‘school based*’, ‘health promotion*’, and ‘cancer awareness*’ were combined using the Boolean terms ‘OR’ and ‘AND’, Medical Subject Headings (MeSH), and truncation ‘*’. The search was conducted between January 2010 and March 2022 and updated in April 2024. The complete search strategy is presented in (SI B).

### Study selection

All identified papers were exported to Covidence, an online software package [64], for screening. Duplicates were deleted automatically in Covidence. During title and abstract screening and subsequently full text review, papers were independently screened against the inclusion and exclusion criteria for suitability in a two-stage process by the authors (S.L., S.F., J.H., and M.M.S.). Screening discrepancies were resolved by a third independent reviewer. Reference lists of eligible papers were checked for additional studies that potentially met the inclusion criteria.

### Assessment of quality

The research design guided the choice of the quality appraisal tool. The quality of all the included studies was assessed using the Mixed Methods Appraisal Tool (MMAT) [65]. This tool helps appraise the methodological quality of five study categories namely: qualitative research, randomized controlled trials (RCTs), non-RCTs, quantitative descriptive studies, and mixed method studies. Quality was assessed by two independent reviewers (S.L. and M.M.S.) using either a ‘yes’, ‘no’, or ‘can’t tell’ vote. Any disagreements that arose between the reviewers were resolved through discussion. All studies, regardless of their methodological quality were included in this review to reduce the risk of study selection bias.

### Data extraction

Data from the included papers were extracted using a standardized table. For a more detailed extraction table please see (SI C). Data were extracted by the first author (S.L.) and then cross-checked by all authors to ensure accuracy (S.F., J.H., and M.M.S.). Data extracted from each article included author(s) and year of publication and study design; aim of the study; sample (including size, age, and gender) and setting; instruments used; timing of assessments; outcomes measured; and findings relating to: overall cancer awareness, knowledge of signs and symptom and knowledge of key risk factors, help and health-seeking for cancer symptoms, barriers and facilitators and help-seeking for cancer symptoms, awareness of and engagement in health risk behaviours linked to cancer. For interventional studies, only pre-intervention data were extracted.

### Data synthesis and integration

Due to the heterogeneity of the study designs, outcomes, instruments, and data collection settings, a meta‐analysis was not possible. A narrative synthesis approach was chosen [66], informed by Popay *et al*. [67]. Studies were tabulated within the data extraction table to allow for initial comparison. Relationships within and between the reviewed studies were explored to identify similarities and differences. Findings were then presented and synthesized narratively according to the review outcomes as follows: (i) cancer awareness; (ii) knowledge of cancer signs and symptoms; (iii) knowledge of cancer risk factors; (iv) help and health-seeking; and (v) health risk behaviours.

## Results

### Study selection

**Fig. 1.**
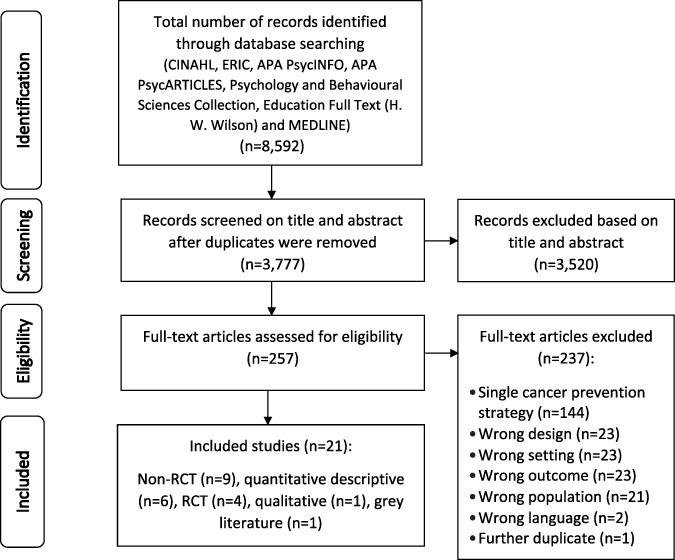
Record identification, screening, and selection process.

Overall, 8592 records were identified through database searching. Following the automated deletion of duplicates, 3777 records were screened based on title and abstract and 3520 irrelevant records were excluded. The full texts of the remaining papers (*n* = 257) were screened. Papers that did not meet the review eligibility criteria (*n* = 237) were excluded and the remaining 20 papers were included in this review. One further study was identified from hand searching. Therefore, a total of 21 studies were included in this review. The study identification, screening, and selection process is presented using the PRISMA flow diagram [57] in Fig. 1.

### Methodological quality

All the included studies had clear research questions(s). The only included qualitative study [38] met all five quality appraisal criteria. Of the included RCTs (*n* = 4), it was difficult to tell if outcome assessors were blinded to the intervention provided in three studies [33–35, 37]. The seven cross-sectional quantitative studies met most MMAT criteria; though, three studies did not report whether confounders were accounted for in the design and analysis [29, 32, 39, 42, 43, 45, 48]. All 21 studies addressed the sampling strategy, except for three quantitative non-RCTs [36, 40, 41]. Validity and reliability of instruments were reported in 11 studies [29–32, 34, 36, 37, 39, 42, 43, 48]. A summary of the quality assessment can be found in (SI D).

### Study characteristics

Of the reviewed studies, nine were conducted in Europe. Designs varied across the 21 included studies, including pre-post-test (*n* = 9), surveys (*n* = 7), RCTs (*n* = 4), and a qualitative study (*n* = 1). The sample sizes ranged widely from 75 [38] to 2173 [37] participants. Study characteristics are presented in Table I.

**Table I.** Key characteristics of the included studies

Author(s), year, and country Study design	Sample setting	Outcomes measured
Hudson *et al*. (2023) USA Pre–Posttest	Total adolescents (*n* = 223) Middle and high school	Effects of students’ cancer literacy following a three-part education curriculum and a 10-question survey were applied pre and post each lesson.
Moskal *et al*. (2023) Poland Pre–Posttest	Total adolescents (*n* = 1500) High schools	Nine multiple choice questions on students’ knowledge of common cancers among men and women, age range, risk factors, symptoms, and knowledge about chosen types of cancer.
Kerschner *et al*. (2022) USA Pre–Posttest	Total adolescents (*n* = 521) Public school	Assessing cancer knowledge, fear and fatalism, risk behaviours, cancer-related communication and what/ students gained or did not gain from the course.
Abraham *et al*. (2021) USA Survey	Total adolescents (*n* = 233) Middle high schools	Knowledge of cancer and cancer prevention, including cancer-related health behaviours.
Sugisaki *et al*. (2021) Japan Survey	Total adolescent (*n* = 8701). Elementary, Junior high, and high School	Close-ended multiple-choice questions; exploring impression about cancer and questions including: Do you think you will get cancer in the future? Can cancer be prevented? Can cancer be cured with treatment? In the future will you undergo cancer screening tests?
Hudson *et al*. (2020a) USA Pre–posttest	Total adolescents (n = 164) High school and middle schools	Pre- and posttest cancer literacy multiple choice survey to explore knowledge of cancer, types of cancer, common risk factors, lifestyle factors, cancer statistics and cancer research.
Hudson *et al*. (2020b) USA Pilot Pre–posttest	Total adolescents (*n* = 349) Middle and high schools	Pre- and posttest cancer literacy multiple choice survey to explore knowledge of cancer, types of cancer, common risk factors, lifestyle factors, cancer statistics and cancer research.
Yildirim Usta and Ateskan (2020) Turkey Cross-sectional	Total adolescents (*n* = 275) Private schools	Cancer survey exploring demographics, levels of knowledge, attitudes, and interests in regard to cancer.
Russell *et. al*. (2020) France Pre-posttest	Total adolescents (*n* = 134) Local middle and high schools	Questionnaire assessing beliefs regarding cancer risk, preventative factors, cancer understanding, cancer fatalism and empowerment in relation to cancer (On site visit to the Hygèe Lab; an interactive lab providing information on cancer prevention and treatment).
Al-Azri *et al*. (2019) Oman Cross-sectional	Total adolescents (*n* = 481) Government schools	Cancer Awareness Measure (CAM) tool.
Woodgate and Busolo (2017) Canada Qualitative Ethnography study	Total adolescents (*n* = 75) Junior high or middle school	Qualitative ethnography study that sought to understand adolescent’s conceptualisation of cancer and cancer prevention.
Hubbard *et al*. (2016) Scotland Cluster randomized controlled trial.	Total adolescents (*n* = 2173) State high schools	CAM to explore cancer awareness and help seeking. Cancer Communication tool asking if they have spoken to their mother, father, or someone else about cancer in the previous 2 weeks.
Garcidueñas *et al*. (2015). Mexico Longitudinal pre–post design	Total adolescents (*n* = 831) Middle schools	Knowledge assessment tool (44 questions) on the prevention of breast and cervix cancer. Students survey to investigate mothers’ gynaecological and obstetric history.
Adamowicz *et al*. (2015) Poland Pre-post-test with control group	Total adolescents (*n* = 305). High schools	Cancer knowledge and a diagnostic survey to evaluate health behaviour Inventory.
Stözel *et al*. (2014) Germany Experimental pre–post design.	Total adolescents (n = 235) Vocationally orientated secondary schools	Pre- and Posttest questionnaire, Questions on six health related behaviours. Multiple-choice items with three response options and a four-point response scale to assess intention to engage in protective behaviour.
Heuckmann *et al*. (2014) Germany Survey	Total adolescents (*n* = 369) High school	25 item questionnaires on interest in and attitude towards cancer.
Lana *et al*. (2014) Spain & Mexico Randomized controlled trial.	Total adolescents (*n* = 2001) Secondary education schools	Pre-test online questionnaire, on the presence of six cancer risk behaviours; smoking, unhealthy diet, alcohol consumption, obesity, sedentary lifestyle, and sun exposure.
Kyle *et al*. (2013a) UK Cross-sectional	Total adolescents (*n* = 478) British and Scottish schools	CAM and questions from the Health Behaviour in School-aged Children study relating to alcohol consumption, smoking, sunbed use, sun protection and physical activity.
Kyle *et al*. (2013b) UK and Scotland A controlled before and after study	Total adolescents (*n* = 422) British and Scottish schools	CAM
Kyle et al. (2012) UK and Scotland Cross-sectional	Total adolescents (*n* = 478) British and Scottish schools	CAM

* Cancer Awareness Measure (CAM)

Cancer awareness was measured using a variety of instruments, mainly using questionnaires/surveys [29–37, 39–49]. Cancer awareness was also assessed using researcher-designed questionnaires (*n* = 2). Questionnaires/surveys were designed by professionals with experience in cancer care (*n* = 3). In a study by Abraham *et al*. [43], questions were adapted from a youth risk behaviour survey, exploring adolescents’ perceptions and knowledge of cancer and cancer prevention including cancer related behaviours [43]. A knowledge assessment tool was designed by Calderón-Garcidueñas *et al*. based on short clinical cases that explored knowledge about risk factors, aetiology and preventive measures of breast and cervical cancers [36]. Heuckmann and Asshoff used a survey to examine adolescents’ connection between cancer and a particular risk factor [32]. The same questionnaire was used in a study by Yildirim Usta and Ateskan [42].

Of note, five studies used the same cancer awareness measurement tool (CAM) [29–31, 39, 48], which includes nine sections with 47 items assessing cancer warning signs, cancer risk factors, seeking help, and barriers to seeking help. Kyle *et al*. used a modified version of the cancer awareness measurement tool CAM [68] and questions from the health behaviours of school-aged children (HBSC) to assess health risk behaviours [30]. Six studies used surveys which had been used in previous research [33, 35, 37, 40–42] that incorporated cancer knowledge and cancer risk behaviours (i.e. smoking, unhealthy diet, alcohol consumption, obesity, and sun exposure). One study developed a survey from scientific literature [44] and one qualitative study used focus groups to elicit participants’ perspective of cancer using cancer metaphors [38]. Of the 21 included studies, four assessed knowledge about cancer using a multiple-choice question format [34, 40, 41, 45]. The studies captured adolescents’ knowledge of cancer by examining their knowledge of cancer signs and symptoms [29, 31, 37, 39, 48] risk factors [29, 30, 32–34, 37, 39–44, 46–49] and help-seeking [29, 31–34, 37, 39, 46–49].

### Cancer awareness

Cancer awareness was not specifically defined within the studies. Studies measured cancer awareness as a construct; however, they did not report on a total score for cancer awareness. Measures of cancer awareness were based on examining adolescents’ knowledge of cancer signs and symptoms [29, 31, 37–40, 44, 46–49] and risk factors [29, 30, 32–34, 37, 39–44, 46–49]. Adolescents’ ‘cancer awareness’ was reported as ‘low’ in six studies [29, 30, 35, 39, 46, 48], ‘poor’ in one study [39], and ‘basic’ in another study [43]. The knowledge items included in the studies assessed the level of information adolescents had about cancer. Adolescents reported that developing cancer is linked to genetics [39, 48], cancer is a disease caused by mutations [40, 41], and participants recognised ‘stage’ as a medical term used to describe how far cancer has spread [43].

### Knowledge of cancer signs and symptoms

Over half of the reviewed studies (*n* = 16) explored adolescents’ knowledge of cancer signs and symptoms. One in five (23.8%) adolescents in a UK study reported not knowing a cancer warning sign [31].However, two studies reported that more than half of the study sample could recognise at least one sign or symptom of cancer [37, 39]. In terms of the most recognised potential warning signs, the majority reported ‘lump or swelling’ as a potential cancer symptom [30, 31, 37, 39, 48], followed by ‘changes in appearance of a mole’ [30, 31, 39, 48]. The least reported sign was ‘a sore that does not heal’ [29–31, 48]. Four studies (*n* = 4) reported that adolescents who knew someone with cancer had higher understanding and knowledge of cancer warning signs [29, 31, 45, 48]. In two UK studies, adolescents held misconceptions about cancer and reported ‘hair loss’ as a cancer warning sign [29, 31].

### Knowledge of cancer risk factors

Most studies (*n* = 16) explored knowledge of cancer risk factors. The knowledge items assessed adolescents’ level of understanding about known cancer risk factors in relation to smoking any cigarettes, drinking more than 1 unit of alcohol a day, low fruit/vegetable intake, eating red or processed meat once a day or more, being overweight, getting sunburnt more than once as a child, HPV infection and doing <60 min of moderate physical activity five times a week. The most frequently known reported risk factors among adolescents included ‘smoking cigarettes’ [30, 32, 37, 39–44, 48], followed by ‘drinking alcohol’ [34, 39, 48], ‘unhealthy diet’ [33, 40, 43], and ‘exposure to sun’ [32, 33, 42, 44, 48]. However, there was uncertainty amongst adolescents about certain cancer risk factors. The least reported risk factors for cancer were ‘not eating enough fruit and vegetables’ [30, 34, 39, 48] and ‘eating red or processed meat’ [39, 44, 48]. In a study in Oman, 63.2% of adolescents reported a change of lifestyle as the most reported contributing risk factor [39]. Three studies reported adolescents’ misconception of risk factors [32, 42, 44].

In a German study, adolescents did not recognise ‘smoking the hookah’ (70.2%), ‘drinking alcohol’ (55.8%) and being ‘overweight’ (18.7%) as cancer risk factors. They incorrectly reported ‘contact with cancer patients’ (1.4%), ‘frequent common cold’ (1.6%) ‘hypertension’ (6%) as risk factors, and 68.5% believed that cancer was unrelated to age [32]. Likewise, in a Turkish study, adolescents incorrectly reported that having ‘numerous birthmarks’ (47.8%), ‘hypertension’ (26.1%), ‘frequent common cold’ (15.7%), and contact with ‘other cancer patients’ (9.5%) were all cancer risk factors [42]. Similarly, in a French study, only 24.8% of adolescents recognised that frequent consumption of ‘red meat’ was understood as a risk factor, only 5.5% reported frequent consumption of ‘fruits and vegetables’ as a protective factor, and only 14.2% of adolescents thought ‘regular physical activity’ had no influence on cancer risk [44].

### Help and health-seeking

Seeking information and eventually help for cancer were assessed in 10 studies (*n* = 10) [29, 31, 32, 37, 39, 42, 43, 45, 47, 48]. The knowledge items included in the studies assessed adolescents’ understanding on where to find help regarding cancer. Adolescents suggested seeking information about cancer from Google and other websites [43], followed by discussion with parents [42, 43], friends [42], doctors [43], and people affected by cancer [42]. Online videos, educational video games, and educational websites were reported as being the most helpful sources to learn about cancer and cancer prevention [43].

Adolescents indicated that they would seek medical help within 3 days [29], and 2 weeks for a symptom they thought might be cancer [39, 48]. In a study by Sugisaki *et al*. [45], adolescents suggested that they would partake in cancer screening in the future. Of the six studies which explored barriers and facilitators to help-seeking for cancer symptoms [29, 31, 32, 37, 39, 48], the most common emotional barrier identified was ‘worry about what the doctor might find’ [29, 31, 37, 39]. The most reported service-level barrier was being ‘difficult to make an appointment’ [39] and ‘difficulty talking to the doctor’ [29, 48]. The most common practice-level barrier was being ‘too busy’ [29, 39]. The least endorsed barrier among adolescents was ‘difficulty arranging transport’ [29, 39]. Facilitators to help-seeking were not reported in the included studies.

### Health risk behaviours pertaining to cancer

Most studies (*n* = 15) explored health risk behaviours linked to cancer either by (i) eliciting adolescents’ own understanding of health risk behaviours linked to cancer or (ii) exploring adolescents’ self-reported engagement in health risk behaviours. Overall, studies found uncertainty regarding recognition levels of health risk behaviours pertaining to cancer among adolescents [30, 32–34, 40, 41, 43, 44, 47].

Adolescents lacked awareness of health risk behaviours which increase the risk of cancer including ‘obesity/overweight’ [30, 32, 33, 42, 43, 48], ‘low uptake of physical activity’ [30, 39, 44, 47, 48], ‘eating fewer fruit and vegetables’ [30, 37, 39, 48], ‘eating red or processed meat’ [30, 39, 48], ‘alcohol use’ [32, 33, 43, 48], and ‘sun exposure’ [30, 39, 43]. Adolescents also stated that cancer prevention is important, and protective lifestyle factors such as increasing water intake, consuming more fruit and vegetables, and increasing activity levels play an important role in the prevention of cancer at a young age [42–44].

Recent studies in the UK and the USA reported diverse health risk behaviours among adolescents [30, 43]. The USA study found that adolescents engaged in positive health practices, with 93.1% drinking at least one glass of water daily, 87.1% abstaining from alcohol in the past month, and 76.4% consuming fruits and vegetables daily. However, just over half of participants (56.2%) met recommended exercise levels, and less than half (45.1%) used sunscreen regularly [43]. The USA study also noted that 12.8% of adolescents reported e-cigarette or tobacco use, with 5.2% specifically using tobacco [43].

The UK study also highlighted some concerning trends, with 7.5% of adolescents identifying as current smokers, 15.1% consuming alcohol weekly and significant proportions neglecting sun protection, with 36% never using sun cream. Additionally, 59.8% of UK adolescents exercised less than four times a week [30].

## Discussion

Overall, cancer awareness among adolescents was low [29, 30, 35, 39, 46, 48]. There appears to be a scarcity of directly comparative literature among the adolescent population. Much of the existing evidence has been generated from research with adults, thus limiting its transferability to the adolescent population. Our findings of low cancer awareness are comparable to previous findings of a survey of adults aged 50 years in Australia, Canada, Denmark, Norway, Sweden, and the UK using the Awareness and Beliefs about Cancer measure [28]. Forbes *et al*. identified that adults in the UK had low awareness of age-related risk and the highest perceived barriers to symptomatic presentation, but symptom awareness in the UK did not differ from other countries [28]. Respondents from Denmark had higher awareness of age-related risk and few perceived barriers to symptomatic presentation.

Lifestyle factors have been shown to strongly influence the risk of cancer with 30–50% of all cancer cases being preventable [69]. It appears from this review that adolescents are under informed about healthy lifestyle habits which mitigate modifiable cancer risk factors [29–34, 37, 39–44, 48]. This has the potential to lead to a higher level of engagement in risk health behaviours [30, 32, 33, 37, 39, 41–43]. Several studies have investigated knowledge, attitudes, health risk behaviours, and delay in presentation in the adult population [70–72], while evidence relating to adolescents is developing [29–49].

Adolescents with inadequate cancer awareness are less likely to recognise cancer signs and symptoms and participate in preventive measures such as making healthy lifestyle choices. In addition, researchers have found that low awareness of potential cancer symptoms is associated with patient delay in help-seeking for cancer [70]. Other potential influences on help-seeking include negative beliefs about cancer outcomes [38, 42, 45], barriers to symptomatic presentation [29–31, 37, 39], and poor awareness of health risk behaviours linked to cancer [29–31, 37, 39–44].

Adolescents can take appropriate actions regarding lifestyle choices both, at a young age and where necessary later in life. Increasing cancer awareness among adolescents gives them the ability to understand healthcare information and make appropriate health decisions. Several studies indicate that education about cancer and associated risk factors may be one way to encourage protective behaviours among adolescents [34, 37, 41]. Providing education on the potentially negative impact of smoking, excess alcohol consumption, poor diet, sedentary lifestyle, and sun exposure can help increase knowledge on cancer prevention behaviours [32–34, 37, 40, 41, 43, 44].

Evidence suggests that interventions can improve adolescents' help-seeking behaviour by increasing cancer communication and self-efficacy [31, 33–37, 40]. The adoption of positive lifestyle choices at a young age is fundamental to reducing the risk of developing cancer later in life [29, 34, 35, 37, 39, 40]. The literature suggests that there are insufficient educational programmes, which emphasise the influence of lifestyle on cancer morbidity and mortality [29, 31–35, 37, 39, 43]. The period of adolescence is the ideal age for health education, when a person cognitively understands the consequences of engaging in health risk behaviours [32, 36, 40]. Secondary schools are an ideal setting to provide education on cancer awareness and health risk factors associated with developing cancer later in life [36, 37, 44]. While school-based educational programmes are important, a multi-faceted, collaborative approach involving various stakeholders is recommend for effective cancer education and prevention among adolescents. This includes using diverse teaching methods such as expert talks, videos, and hands-on activities [43]. Collaboration with healthcare providers and community organisations is recommended to support such programmes [43]. Some initiatives also involve parents to reinforce behaviour change beyond the school setting [73].

The long-term effects of such educational programmes beyond adolescence are poorly understood. Indeed, the effectiveness of existing cancer awareness interventions for adolescents is assessed in the short term, ranging from 2 weeks post-intervention up to 24 weeks [31]. In addition, the implementation of health education programmes for cancer prevention during adolescence faces several barriers. These include but are not limited to: inadequate resources such as staff, time, and funding [74]; adolescent and family-related factors such as low motivation, schedule conflicts, and fatigue [75]; negative emotions associated with cancer, such as sadness and fear; a lack of personal connection to cancer [76]; educational barriers like misinformation and low health literacy [77]; and implementation difficulties such as poor follow-up and addressing sensitive topics like obesity [18].

Longer follow-up is necessary to determine the long-term effect of such interventions. In addition, some cancer awareness programmes may not be sufficient to elicit sustained changes in cancer knowledge, attitudes, and behaviours. Therefore, frequent, repeated interventions may be necessary to ensure that the targeted educational approach continues to have the desired sustained effect.

## Limitations

To our knowledge, this is the first mixed-method systematic review on the topic of cancer awareness among adolescents. The results of the review should be interpreted considering its limitations. Some of our inclusion criteria may have led to a narrower perspective on the topic area. This review was limited to English-only publications. Grey literature such as unpublished research was excluded, increasing the risk of study selection bias. In addition, studies from low and very low HDI and consequently lower resource countries were not included. A lot of the findings, however, are transferable to countries ranking low and very low on the HDI, given the high cancer incidence and prevalence in these countries. Added is the need for cost-effective interventions including, for example, mass cancer awareness and education camps, community health worker and navigator-based care delivery models, and mobile technology for outreach and education [78].

## Conclusion

Findings from this review of 21 studies suggest that cancer awareness among adolescents is low. However, variation was seen across the included studies with the reasons for such variation being unclear. Low cancer awareness among adolescents represents a lost opportunity to maximise the potential of preventing cancer and promoting early diagnosis. There is a need for further research to assess and explore, in-depth, adolescents’ awareness of cancer signs and symptoms, cancer risk factors, and perceived barriers to seeking medical advice.

Providing cancer education can help adolescents better understand cancer signs and symptoms and improve health- and help-seeking behaviours. Messages about cancer need to be targeted and tailored to the adolescent population to prevent the development of health inequalities later in life; such tailoring requires an understanding of context.

It is recommended that future studies clearly articulate the theoretical and operational definitions of cancer awareness and use standardised tools to measure cancer awareness. In addition, longitudinal research is needed to provide a clearer understanding of the association between cancer awareness, knowledge of risk factors, health risk behaviours, and help-seeking among adolescents as well as outcomes such as cancer diagnosis, stage at diagnosis, treatments received, and mortality.

## Supplementary Material

cyaf014_Supp

## Data Availability

The data that support the findings of this review has been supplied as supplementary files and is taken from the included studies which are publicly available.
