# Supplementary material for: Cancer awareness among adolescents in second-level education: a mixed methods systematic review
Source: Health Educ Res. 2025 May 12;40(3):cyaf014. doi: 10.1093/her/cyaf014 (PMC12068056; doi:10.1093/her/cyaf014)
Supplement: cyaf014_Supp [file cyaf014_Supp.zip › Supplementary File SI B Search strategy.docx]

**Supplementary Table A1** Search strategy used in searching the databases

| Adolescen* OR teen* OR “young people” OR “young person*” OR “young adult*” OR child* OR youth* OR young* OR kid* OR student* OR pupil*  AND  Cancer* OR oncolog* OR tumo* OR malignan* OR neoplas*  AND |
| --- |
|  |
| Aware* OR know* OR educat* OR attitude* OR belie* OR health N5 aware* OR health N5 know* OR health N5 educat* OR health N5 attitude* OR health N5 belie* OR health N5 risk* OR health N5 seek* OR “help seek*” OR “help-seek*” OR cancer N5 screen* OR cancer N5 risk* |
|  |
| AND |
|  |
| School* OR "school based" OR "middle school*" OR "elementary school*" OR "high school*" OR highschool* OR college* OR secondary OR “second level” |
|  |
|  |
