# Supplementary material for: Cancer awareness among adolescents in second-level education: a mixed methods systematic review
Source: Health Educ Res. 2025 May 12;40(3):cyaf014. doi: 10.1093/her/cyaf014 (PMC12068056; doi:10.1093/her/cyaf014)
Supplement: cyaf014_Supp [file cyaf014_Supp.zip › Supplementary File SI C Long extraction.docx]

**Supplementary File Table SI C** Long extraction

| **First Author**  **(Year)**  **Country**  **Study Design** | **Aim** | **Sample (Who, number, gender, age, grade, range, mean)**  **Setting** | **Outcomes measured** | **Overall cancer awareness** | 1. **Knowledge of signs and symptom** 2. **Knowledge of key risk factors** | 1. **Help seeking** 2. **barriers to help seeking** 3. **Facilitators to help seeking** | 1. **Health risk behaviours relating to cancer** 2. **Other** |
| --- | --- | --- | --- | --- | --- | --- | --- |
| Hudson et al. (2023)  United States  Pre-Posttest | To increase cancer literacy in the region | adolescents  (n=223) male (44.84%) (n= 100); female (n=123) (55.16%)  Middle and high school. 8^th^ to 12^th^ grade. | Survey based on the effects of student’s cancer literacy following a three-part education curriculum and a 10 question pre and l 10 question posttest. | Increased adolescents’ cancer literacy. | 1. 32.9% noted a benign tumour is cancerous, 64.5% know when cancer has metasized, 40.0% reported how cancer cells differ than regular cells, 53.9% stated know the types of cancer changes after cancer has metasized. 2. 74.3% know common risk factors, 75.8% know lifestyle choices that increase one’s chance of developing cancer, 44.4% know ways that infectious diseases can increase cancer risk, 80.3% stated what risk factors to avoid if trying to decrease their cancer risk. 46% suggested regular exercise decreases the risk of obesity and as a result, increases the risk of developing cancer. | 1. NR 2. NR 3. NR | 1. NR 2. 47.1% report what immunosuppression is,   40.8% report why women are receiving hormone therapy at a higher risk of developing cancer. 70.4% reported being aware of what is *not* a cancer treatment,  25% report what a stem cell is,  51.3% reported being aware of the disadvantages of combination therapy, 72.6% report the most common types of cancer treatments, 12% report what combination therapy is (combining at least three drugs), 49.4% report radiation therapy, 20.0% report hormone therapy is only used for breast, prostrate, and liver cancer patients. 38.5% reported surgeons sometimes decide to remove only some of a cancerous tumour as opposed to the whole tumour. 13.7% reported only in certain scenarios would precision medicine not work. |
| Moskal et al. (2023)  Poland  Pre-Posttest | To improve cancer awareness using and educational campaign | Adolescents (n=1500) Male (48%), Female (52%). Aged 16-19 years.  High schools | Nine multiple choice questions on students’ knowledge of common cancers among men and women, age range, risk factors, symptoms, and knowledge about chosen types of cancer. | Mean score of correct answer was 709 (47.3%) | 1. 34.93% reported alarming symptoms of Melanoma, 2. 36.47% reported birth control associated with higher risk of breast cancer,   33.13% reported physical activity reduces risk of breast cancer. 83.73% reported risk factors for lung cancer, 18% report what type of cancer can be triggered by HPV,  34.13% reported risk factors for melanoma. | 1. NR 2. NR 3. NR | 1. 33.13% believed that physical activity reduces the risk of breast cancer. 2. NR |
| Kerschner et al. (2022)  United States  Pre-Posttest | To assess cancer knowledge, fear and fatalism, risk behaviours, cancer-related communication and what did students hope to gain and did gain from the course. | Total adolescents (n=521).  9^th^ to 12^th^ grade.  Aged 14 to 19 years.  Public school | Assessing cancer knowledge, fear and fatalism, risk behaviours, cancer-related communication and what students hope to gain and did gain from course | After the CHEC, overall student cancer knowledge  increased significantly (percent change = 5.87%,  p < 0.0001) | 1. 71.32% of people with cancer always experience signs or symptoms of the disease,   90.64% suggested regular checkups, medical screening tests, and noticing changes in your body may help find early signs of cancer,  67.18%'s stated that recommendations for cancer screening exams are based on a person's age, risk factors and family history,   1. 75.72% reported all girls and boys who are under 11 and 12 reported that they should get the HPV vaccine,   79.96% believed eating a high fat, low fibre diet will help prevent cancer, 60.15% stated that physical activity lowers your chance of getting cancer,  65.44% reported that in order to help prevent skin cancer, people only need sunscreen in the summer months, 88.25% reported tobacco use and/ or poor diet increases your chances of getting cancer, 66.28% stated the HPV vaccine can prevent most cervical cancers in women and most head and neck cancers in men and women.  I believe if someone is meant to get cancer, it doesn’t matter if they eat healthy foods, they will still get (scale maximum 1) (M=.30, SD=.46). | 1. 90.64 reported regular checkups, medical screening tests, and noticing changes in your body may help find early   signs of cancer  when asked how often do you talk with your family or friends about cancer or cancer risk specifically 76.25% reported never/rarely and 23.74% reported sometimes/often/always.   1. NR 2. NR | 1. 91.63% of participants stated that the goal of early detection testing is to find and stop cancer before it grows and spreads 73.11% of participants reported that cancer screening is looking for cancer before a person has any symptoms, 2. 77.18% reported tumors can be benign or malignant, 82.12% stated that cancer cells grow without control or order,   78.46% reported that cell mutation is a change in the cell structure.  70.60% reported that a benign tumor means you have cancer.  87.45% stated that stage is a medical term to describe how far a cancer has spread.  36.38% reported that if someone has breast cancer and it spreads to the liver, the person then has breast cancer and liver cancer.  I believe that if someone is meant to have cancer, it doesn’t matter what they eat, they will get cancer anyway (scale maximum 1) (M=.33, SD=.47). I believe if someone has cancer, it is already too late to do anything about it (scale maximum 1) (M=.12, SD=.32).  I believe someone can smoke all of their life, and if they are not meant to get cancer, they won’t get it (scale maximum 1) (M=.24, SD=.43).  I believe if someone is meant to get cancer, they will get it no matter what they do (scale maximum 1) (M=.28, SD=.45).  I believe if someone is meant to get cancer, it was meant to be (scale maximum 1) (M=.22, SD=.41).  I believe if someone gets cancer, their time to die is near (scale maximum 1) (M=.17, SD=.37).  I believe if someone gets cancer, that’s the way they were meant to die (scale maximum 1) (M=.10, SD=.30).  I believe that getting checked for cancer makes people think about dying (scale maximum 1) (M=.56, SD=.50).  I believe if someone is meant to get cancer, they will have cancer (scale maximum 1) (M=.25, SD=.43).  I believe some people don’t want to know if they have cancer because they don’t want to know they may be dying from it (scale maximum 1) (M=.81, SD=.39).  I believe if someone gets cancer, it doesn’t matter when they find out about it, they will still die from it (scale maximum 1) (M=.16, SD=.37).  I believe if someone gets cancer, a lot of different treatments won’t make any difference (scale maximum 1) (M=.19, SD=.40).  I believe if someone was meant to have cancer, it doesn’t matter what the doctor tells them to do, they will get it anyway (scale maximum 1) (M=.17, SD=.38).  I believe cancer will kill most people who get it (scale maximum 1) (M=.61, SD=.49). |
| Abraham *et al.* (2021)  United States  Survey | Explore adolescents’ perceptions of cancer and cancer risk factors and identify preferences for receiving cancer education. | Adolescents (n=233), Male (48%), Female (52%).11^th^ and 12^th^ grade  Middle and high schools (n=3). | Survey based on health belief model,  the youth risk behaviour survey  and the cancer,  clear & simple (CC&C) curriculum. | Overall authors note “a basic understanding of cancer knowledge.” The need for an increase in cancer knowledge, specifically on how they control their cancer risk and prevention strategies, through game-based education to the on cancer-related topics. | 1. 82% reported cancer is not just one disease; 89% identified cancer cells grow without control or order. 96% recognized “stage” as a medical term used to describe how far cancer has spread. 66% stated a person has some control over their risk of developing cancer. 2. Awareness of cancer risk factors were 91% tobacco, 80% poor diet, 80% physical activity. Less commonly perceived risk factors included alcohol consumption 78% and exposure to sunlight 71%. | 1. 72% source cancer information on google and websites, 68% discuss with parents, 58% discuss with doctors. 2. NR 3. NR | 1. 93.1% reported drinking a glass of water every day, 87.1% did not consume alcohol in the last 30days, 76.4% reported eating one piece of fruit, 73.8% one vegetable, 56.2% got 60 minutes exercise for 5 days, 45.1% used sunscreen, 12.7% reported e-cigarette use, and 5.2% tobacco use. 2. 95% reported cancer prevention was important. 50% currently try to make decisions that will lower their chances of developing cancer. 37.3% stated they knew how to lower their risks 93% suggested they would share knowledge about cancer prevention with their family 92% would share with friends. |
| Sugisaki et al. (2021)  Japan  Survey | Cancer awareness and understanding of students who have close relatives with cancer | Adolescent (n=2135), Male (n=1060), Female (n=1075), elementary,  (n=2902) Male (n=1481), female (n=1421 girls) junior high,  and (n=3664) Male (n=1526) Female (n=2138)  High School | Cancer questionnaire. | Students having parents or relatives had higher level of awareness. | 1. NR 2. NR | 1. 76.2% stated that they would get cancer screening in the future (student group who reported having a family member with cancer); while 68.3% (other group). stated that they would get cancer screening in the future. 2. NR 3. NR | 1. NR 2. 45.8% reported cancer is preventable, (student group who reported having a family member with cancer); while 49.3% (from the other group, reported cancer is preventable. 22.6% (student group who reported having a family member with cancer) stated they would get cancer in the future; while 11.7% (from the other group), reported they would get cancer in the future. |
| Hudson et al. (2020a)  United States  Pre-posttest | To assess a cancer education intervention and measure cancer health literacy among adolescents | Adolescents (n=164), Male (40.9%), Female (59.2%).  Middle and high schools (n=6) | Cancer literacy survey.  Demographics including a  10-item pretest and posttest survey.  Post intervention. | Overall test score | 1. 87.7% agreed cancer is a disease caused by mutations, 44.4% suggested a benign tumor is cancerous 59.9% understood what metastasized meant, 1.2% identified the two major types of cancer, 58.1% understood biopsy. 2. 75% identified smoking, unhealthy diet and risky behaviours as cancer risk factors. 77.2% agreed age, carcinogens environmental risk factors, obesity, and viruses/infectious agents. | 1. NR 2. NR 3. NR | 1. NR 2. 84.5% identified that cancer can impact populations. |
| Hudson *et al.* (2020b)  United States  Pilot pre-posttest | To pilot a cancer education intervention and assess cancer health literacy | Adolescents (n=349), Male (31.3% Female (68.7%).  Middle and high schools (n=4) | Cancer literacy survey  Demographics including a  10-item pretest and posttest survey.  Post intervention. | Marked items for cancer literacy skills. | 1. 93.4% agreed cancer is a disease caused by mutations, 62.5% suggested a benign tumor is cancerous 70.6% understood what metastasized meant, 72.3% understood biopsy.78.7% agreed age, carcinogens environmental risk factors, obesity, and viruses/infectious agents. 2. 78.7% identified smoking, unhealthy diet and risky behaviours as cancer risk factors. 82.2% identified lifestyle changes that increased likelihood of cancer. | 1. NR 2. NR 3. NR | 1. NR 2. NR |
| Yildirim Usta & Ateskan  (2020)  Turkey    Cross sectional | To investigate adolescents’ knowledge on risk factors for cancer, their interest in the disease and affective and behavioral attitudes towards cancer | Adolescents (n=275), Male (44% Female (56%).  Three private schools in Central Turkey and one private school in Eastern turkey (n=4) | Survey based on four parts.  demographics, level of knowledge  about cancer risk factors,  attitudes towards cancer  and students’ interest in cancer. | The need for an increase in developing cancer educational programme with specific focus on early recognition of cancer symptoms, current cancer treatments, and adoption of healthy lifestyle habits. | 1. NR 2. 80.% of participants identified carcinogenic risk factors; going to the solarium often 80.9%, smoking 86.1%, smoking the hookah 77.7%, radioactive 89.1%, ultraviolet radiation 87.9% and x-ray radiation 82.8%, drinking alcohol 66.7%, exposure to sunlight 76.9%, overweight 39.7% and consuming drugs 69.1%., 47.8% reported numerous birthmarks, 26.1% hypertension, frequent common cold 15.7% and contact with other patients 9.5%. | 1. 25.1% agreed and strongly agreed that getting cancer would motivate them to speak with friends about anxieties, 29.6% would talk to parents and 42.5% would talk with affected persons. 2. NR 3. NR | 1. NR 2. In total, 69% agreed or strongly agreed that the idea of getting cancer motivates them to a healthier life, 57.8% agreed or strongly agreed that getting cancer motivates them to change their life and 50% would seek information on cancer. 65.9% of participants either agreed or strongly agreed that they feel sad when thinking about cancer. 11.6% have negative thoughts about cancer, 14.2% thinking about cancer makes them anxious, 18.4% feel uncertain faced with the idea of cancer, 14.4% feel worried when thinking about cancer, 14.5% cancer makes them feel scared and 58.8% feel depressed when thinking about cancer.51.1% would donate to a cancer charity and 69.3% motivates them to communicate with children whom have cancer. |
| Russell et. al. (2020)  France  Pre-Posttest | To assess the impact of a visit to a cancer prevention centre exhibition | Adolescents (n=134)  Male (n=39.1%)  Female (60.9%)  Local middle and High schools | Questionnaire assesses beliefs.  regarding cancer risk,  preventative factors,  cancer understanding,  cancer fatalism  and empowerment  in relation to cancer  (Baromètre Cancer). | Adolescents’ cancer understanding - adolescents.  underestimate the role of alcohol and, tobacco as cancer risk factors. | 1. NR 2. Cancer understanding on a 5-point agreement scale; If childhood sunburns are well treated, they have no consequences in adulthood 2.94; strong alcohol is bad for your health 3.31, breathing city air is as bad for your health as smoking cigarettes 3.40, how many cigarettes a day does it take to increase the risk of cancer (1;2-9, 9-19>20) (1.67). | 1. NR 2. NR 3. NR | 1. NR 2. Mean values on 5-point scale; Felt informed about cancer 3.46, capable to act against cancer 3.32. Regular Cancer screening can pre-empt heavy treatment in case of disease 3.82, Road accidents kill more people than cancer does 3.16, |
| Al-Azri  *et al.* (2019)  Oman  Cross- sectional | To identify knowledge of cancer risk factors, symptoms, and barriers to seek medical help | Adolescents (n=481), Males (n=258, 53.6%), Female (n=223,46.4%). 15-17years,  Government schools (n=6) | Cancer Awareness Measure tool (CAM)  (Cancer Research UK). | Recognition  of cancer risk factors and symptoms was low | 1. The average recognition level of all cancer symptoms was 39.6%. with 70.1% reported lump or swelling as a cancer symptom; 54.3% changes in appearance of a mole; 45% unexplained pain; 41% unexplained bleeding; 39.9% a sore that does not heal; 38.3% unexplained weight loss; 36.2% loss of appetite, 28.7% change in bowel/bladder habits; 21.4% persistent cough/hoarseness and 20.4% persistent difficulty in swallowing. 8.9% genetics; 12.9% environmental factors, 1.2% aging; 63.2% lifestyle and 3.7% purely by chance. 2. 79.8% recognised smoking as a risk factor; 67.8% drinking alcohol; 55.7% second hard smoking; 41.4% sunburn; 39.5% being overweight; 30.4% HPV infection; 26.8% infection with Hepatitis B or C; 28.9% inactivity and 20.2% eating less than 5 fruit/vegetables a day, 12.1% eating red processed meat once a day and also agreed that a change in lifestyle is a contributing risk factor in developing cancer; and 23% having a close relative with cancer. | 1. 88% would seek help for cancer symptoms within 2 weeks. 2. 66.9% worry about what the doctor might find; 58% too scared; 42.8% not confident to talk to a doctor; 42.4% too embarrassed; 59.9% too busy; 48.4% too many things to worry about; 42% difficult to arrange transport; 44.7% difficulty making an appointment, 41.4% doctor was difficult to talk to; 18.7% worry about wasting the doctor’s time. 3. NR | 1. NR 2. 84.4% agreed cancer can be cured if detected early. |
| Woodgate & Busolo (2017)  Canada  Qualitative Ethnography study | “To understand  adolescents’ conceptualisation of cancer and  cancer prevention” (p.1) | Adolescents (n=75), Males (n=22, 26.7%), Female (n=53, 73.3%).11-19 years;(Mean age 14.5 years, (SD=2.1),  Junior high or middle school (n=6) | Open-ended interviews,  photovoice and  focus groups. | Adolescents use metaphors to describe and make sense of cancer. | 1. NR 2. Adolescents reinforced the importance of building a strong healthy body (strong soldier metaphor) in order to be better prepared to fight the dreaded invading cancer cells and shared photos of factors and activities (e.g., exercising and eating fruits and vegetables; that contributed to being strong and healthy. | 1. NR 2. NR 3. NR | 1. NR 2. Adolescents held misconceptions about cancer (non-Caucasian felt sun tanning was not harmful to because of brown skin and adolescents’ belief that cancer, as being equated with death, having little chance of survival, almost no cure and is part of God’s will. |
| Hubbard *et al*. (2016)  Scotland  Cluster Randomized controlled trial. | To identify if a psycho-educational intervention increased adolescents’ cancer awareness and addressed medical help-seeking. | Adolescents, (n=2173), Male (n=1071, 49.3%), female (n=1102, 50.7%)12-13 years; mean 12.4 years, SD = 0.55),  State high schools (n=20) | CAM (Demographics, Cancer Awareness Measurement. (CAM). Items assessing awareness of cancer warning signs, risk factors, incidence, screening programmes and attitudes towards help  seeking  Cancer.  Recognition of signs and symptoms of cancer was assessed through a nine-item question.  Recognition of cancer risk factors was assessed through an 11-item question. Eleven cancer risk factors were listed. Responses were recorded on a 5-point Likert scale.  Barriers to help-seeking were assessed with 11 items. Communication tool. Adolescents were asked if they have spoken to their mother, father, or someone else about cancer in the previous 2 weeks. |  | 1. 79.8% recognizing lump or swelling, 51.9%, change in bowel habits 48.1%, unexplained bleeding, 43.4%, change in appearance of a mole, 45.6% unexplained pain, 37.9% unexplained weight loss, 30.3% difficulty swallowing, 30.3% difficulty swallowing, 26.1% a sore that does not heal. 2. Mean number of cancer risk factors at Intervention group recognized was 4.2/11 (SD=2.06) and control group 3.8/11 (SD=2.12) 84.1% smoking, 54.8% second hand smoking, 41% being overweight and 41% getting sunburnt, 40.1% excess alcohol consumption, 23% low levels of physical activity, 21.6% being over 70, 14.5% HPV infection and 14.5% eating red/processed meat and 9.9% low fruit/veg consumption. 32.2% having a close relative with cancer. | 1. NR 2. 70.3% worry about what the doctor might find (50.8%, Too scared 45.6% too embarrassed and 50.8% not confident to talk about symptoms. 16.2% have other things to worry about, 15.2% are too busy and 13.6% find it difficult to arrange transport. 29.3% reported difficulty talking to doctor, 29% worry about wasting the doctors time and 20.6% find it difficult to make an appointment. 3. NR | 1. NR 2. Cancer communication 9.1%, reported they would speak to someone about cancer, 7.2 (SD=4.13) reported anxiety. |
| Garcidueñas  *et al*. (2015)  Mexico  Longitudinal pre-post design | To design and implement an educational intervention to develop knowledge and skills of health promotion in relation to cancer | Adolescents (n=831), (Male n=382), Female (n=449) (Mean 13.23 SD=1.08 years)  Middle school | Knowledge assessment tool (44 questions)  on the prevention of breast and cervix cancer.  Students survey to investigate  mothers gynecological and obstetric history. | Improvement in students’ knowledge cancer skill. | 1. Level of overall knowledge (i.e., correct answers) was 53.44% pre-test. 2. NR | 1. NR 2. NR 3. NR | 1. NR 2. NR |
| Adamowicz *et al.* (2015)  Poland  Pre-post-test with control group | To assess the impact of a cancer prevention education programme | Adolescents (n=305), Male (n=133, 43.6%), Female (n=172, 56.4 %)  High school students. | Pre education questionnaire on cancer knowledge and a diagnostic survey to evaluate health behaviour Inventory (HBI), healthy eating habits, disease preventing behaviours, health practices and mental attitude.  Completed 2 and 12 months after the education using 4 teaching methods.  Method 1; 2 x 45-minute presentation and 15 min discussion  Method 2; 2 x 45 min presentation supported by audio visuals and film.  Method 3; write a note on cancer prevention followed by a discussion.  Method 4; Interview with a patient suffering with cancer. | Knowledge among adolescents was low and coexists with low levels of health behaviour inventories. | 1. Knowledge regarding cancer before education was 64.9 with a maximum of 136. 2. NR | 1. NR 2. NR 3. NR | 1. NR 2. NR |
| Stözel *et al.* (2014)  Germany  Experimental pre-post design. | To promote awareness of cancer- related risk factors and increase the intention to engage in protective behaviours | Adolescents (n=235), Male 49%, female, 41%). 12 to 15 years; Mean 13 years, vocationally orientated secondary schools (n=20). | Pre/Posttest questionnaire  Questions on six health related behaviours  engaging in physical activity; applying sun lotion in the summer; smoking cigarettes; eating fruits and vegetables; eating sweets and drinking soft drinks; drinking alcohol, using a three or four-point response scale. There were also multiple-choice items with three response options, as well as intention to engage in protective behavior was assessed. using a four-point response scale.  A risk-score was created based upon a combination of the six adolescent risk behaviors investigated. | Raised awareness and increased knowledge of health promotion intentions. | 1. NR 2. In the CG reported drinking alcohol 65% daily sweet intake 28% insufficient fruit/vegetables 22% physical inactivity 15%, no sun protection 4% and smoking 8% In the IG reported drinking alcohol 53%, daily sweet intake 24%, insufficient fruit/vegetables 15%, physical inactivity 13%, no sun protection 5% and smoking 5%. | 1. NR 2. NR 3. NR | 1. NR 2. At pre-test, intention- to engage in protective behaviour differed between CG and IG showing a higher intention in the IG |
| Heuckmann  *et al*. (2014)  Germany  Survey | To investigate students’ interest in and attitudes towards cancer from a cognitive, effective, and behavioural component. | Adolescents (n=369), 16–18 years; Male 43.9% and female 54.7%; High school (n=8) | 25 item researcher designed questionnaire interest in and attitude towards cancer. | Students’ willingness to deal with the topic cancer.  is highly dependent on their interest, their emotional responses.  and their beliefs about the controllability of cancer. | 1. NR 2. “Going to the solarium often” (rated as carcinogenic by 95.4 %), “excessively ex- posed to sunlight” (92.4 %), “smoking cigarettes” (92.1 %), “radioactive radiation” (90.0 %), “ultraviolet radiation” (86.7 %) and “X-ray radiation” (85.1 %). Students correctly identified non-carcinogenic factors as well, e.g., “contact with cancer patients” (1.4 %), “frequent common cold” (1.6 %) and “hypertension” (6.0 %). However, there was a range of un- certainty by students about certain carcinogenic risk factors which are known to increase the risk of contracting cancer; “smoking the hookah” (70.2 %), “drinking alcohol” (55.8 %) and “overweight” (18.7 %). | 1. Intentions for proactive behaviour towards cancer 4-point scale (mean (SD); The idea of getting cancer motivates the student to talk about it with their parents (2.17 (0.91); the idea of getting cancer motivates the student to gather information about the issue (2.67 (0.86); the idea of getting cancer motivates the student to speak with their friends about their anxieties (2.09 (0.80). Students “think, suffering from cancer is a result of lifestyle” (2.77 (0.87), which indicated that students felt empowered to confront cancer through lifestyle choices up to a certain point.   Females rated stronger negative emotions lead to more readiness to talk about cancer (p≤.001), male students were more likely to decline to communicate about cancer (p≤.001), social engagements towards cancer and (p≤.001), lifestyle choices for proactive behaviour (p≤.05), than females.   1. Faced with the idea of getting cancer I feel depressed (2.95 (.97); Thinking about cancer I have negative thoughts (3.47 (.79); Thinking about getting cancer makes me feel anxious (2.64 (.97); Faced with the idea of getting cancer I feel uncertain (2.77 (.97); Thinking about cancer, I feel worried (2.85 (.93); Thinking about cancer, I feel sad (2.52 (1.00); Cancer diseases make me feel scared (3.13 (.91). 2. NR | 1. NR. 2. Stronger negative emotions lead to reconsider lifestyle choices p≤.001). |
| Lana *et al*. (2014)  Spain & Mexico  Randomized controlled trial. | “To assess the impact of a web-based intervention to reduce cancer  risk linked with smoking, unhealthy diet, alcohol consumption, obesity, sedentary lifestyle and sun exposure.” (p.54) | Adolescents (n=2001), CG (n=987, 49.3%), EG (n=1014, 50.7%) 12 to 15years, Secondary education schools | (Pre-test) online questionnaire  On the presence of six cancer risk behaviors; smoking, unhealthy diet, alcohol consumption  obesity, sedentary lifestyle, and sun exposure. |  | 1. NR 2. Not enough fruit and vegetables was reported by the CG 40.1% and EG1 38.3& and EG2 34.4% respectively, smoking CG .5%, EG1 0% and EG2 1.8%, not enough vegetables CG 26.6% EG1 41.6% and EG2 34.4%, Dietary fat, CG 53.6% EG1 54.5% and EG2 49.1%, Overweight/obesity CG 16.1% EG1 14.3% and EG2 14.3%, alcohol CG 1.8% EG1 0.6% and EG2 2.2%, sedentarism CG 31% EG1 31.8% and EG2 21.9% and sun exposure CG 50.7% EG1 49.3% and EG2 21.9%. | 1. NR 2. NR 3. NR | 1. NR 2. NR |
| Kyle et al. (2013a)  United Kingdom  Cross-sectional | To identify adolescents’ awareness of cancer risk factors and associations with health risk behaviours | Adolescents (n=478 (male (n=250, 52.3%, female (n=228, 47.7%), 11–17 years; (mean; 13.8), (SD; 1.24),  British and Scottish schools (n=4) | CAM (Demographics, Cancer Awareness Measure tool. (CAM) (Cancer research UK) and questions from the health behaviours school aged children survey (HBSC) relating to alcohol consumption, smoking, sunbed use, sun protection and physical activity. | Relationship between cancer risk awareness and risk behaviours among adolescents. | 1. NR 2. 87.8% agreed with smoking as being a cancer risk factor, 59.7% second hard smoking, 58.4% being overweight, 51.9% sun exposure, 46.5%, 41.2% family history, drinking alcohol 31.4% HPV infection, 25.5% low exercise, 21.8% older age, 15% eating red meat, and 6.5% low fruit and vegetable intake. (41% agreed that having a close relative with cancer 22% agreed being over 70 years was a cancer risk factor. Also, recognition of cancer risk factors by gender; There was no statistically significant difference in the mean number of cancer risk factors recognized between male and female adolescents [male; 4.41 SD¼2.15) versus female; 4.41 (SD¼2.01); t (476) ¼0.001, P¼1.000]. Males were statistically significantly more likely to agree that second-hand smoke, low levels of physical activity and being aged over 70 were cancer risk factors. Females were significantly more likely to agree that HPV infection and family history were cancer risk factors | 1. NR 2. NR 3. NR | 1. 7.5% current smokers, 15.1% weekly consumption of alcohol, 10.5% sunbed use, 10.9% use no suncream while sunbathing, 36. % use no suncream, 59.8% use physical exercise less than four times a week. 2. NR |
| Kyle et. Al. (2013b)    United Kingdom and Scotland  A controlled before and after study | To identify the effectiveness of school-based interventions. | Adolescents (n= 422), (male (n=221, 52.4%, female (n=228 47.2%)11–17 years (mean; 13.8), (SD; 1.26),  British and Scottish schools (n=4) | CAM (Demographics,  Cancer Awareness Measurement tool. (CAM)  (Cancer research UK). |  | 1. In the intervention schools 23.8% of adolescents reported not knowing a cancer warning sign (T0), 51.4% unexplained pain, 79% unexplained weight loss, 28.2% sore that does not heal, 39% cough or hoarseness, 52.8% unexplained bleeding, 38.9% difficulty swallowing, 54.4% change in bowel habits/bladder habits, 64.9% change in appearance of a mole, 94.4% lump or swelling. In the control Schools 28.8% of adolescents reported not knowing a cancer warning sign (T0). 37.4% unexplained pain, 32.8% unexplained weight loss, 17.4% sore that does not heal, 19.7% cough or hoarseness, 40.2% unexplained bleeding, 32.6% difficulty swallowing, 56.8% change in bowel habits/bladder habits, 49.2% change in appearance of a mole, 80.2% lump or swelling in the intervention schools, recognition of cancer warning signs at T0: 4.6 [SD=2.20]. In the control group recognition of cancer warning signs T0; 3.7 [SD; 2.02]. 2. NR | 1. NR 2. Intervention group at T0; Emotional barriers- Not confident to talk about symptoms (53%); Worried about what the doctor might find (70.2%); Too scared (56.8%); Too embarrassed (56.8%). Practical barriers- Other things to worry about (22.2%); Too busy (24.9%); Difficult to arrange transport (16.4%). Service barriers- Difficult to make an appointment (23.1%); Worried about wasting the doctor’s time (34.1%); Difficult to talk to doctor (35%). Control group at T0; Emotional barriers- Not confident to talk about symptoms (50%); Worried about what the doctor might find (73.5%); Too scared (49.6%); Too embarrassed (51.5%). Practical barriers- Other things to worry about (12.4%); Too busy (17.1%); Difficult to arrange transport (14.7%). Service barriers- Difficult to make an appointment (15.4%); Worried about wasting the doctor’s time (29.2%); Difficult to talk to doctor (34.6%). 3. NR | 1. NR 2. NR |
| Kyle *et al*. (2012)  United Kingdom and Scotland  Cross-sectional | “To determine adolescents;’ awareness of cancer.” (p.2) | Adolescents (n= 478), (male (n=250, 52.3%, female (n=228, 47.7%) 11–17 years; (mean; 13.8), (SD; 1.24),  British and Scottish schools (n=4) | Cancer Awareness Measure tool (CAM)  (Cancer research UK). | Cancer awareness is low among adolescents. | 1. Recognition of cancer warning signs was 4.28 (SD=2.14) out of 9. 26.21% (1 in 4) did not know a sign or symptom of cancer. 89.3% agreed lump or swelling as cancer sign/symptom, 58.8% changes in appearance of a mole, 53.6% change in bowel habits and 48.5% reported unexplained bleeding, 45.8% persistent unexplained pain, 40.6% unexplained weight loss, 35.6% difficulty swallowing, 31.8& persistent cough, or hoarseness and 23.8% a sore that does not heal. Knowing someone with cancer resulted in higher recognition of cancer warning signs mean 4.26 (SD=2.14) out of 9.14.4% (1 in 7) reported. 2. 68.5% of adolescents believed that cancer was unrelated to age. | 1. 73.5% indicated that they would seek medical help for a symptom they thought might be cancer. 2. 71.8% worry about what the doctor might find, 55.6% too embarrassed, 54.4% too scared and 53.3% not confident in talking to a doctor, 34.1% difficulty talking to a doctor, 32.8% worry about wasting their time, 23% too busy and 15.3% difficulty arranging transport. 3. NR | 1. NR 2. Misconception 14.4% reporting ‘hair loss’ as a cancer warning sign |

*Control group (CG), *Experimental group 1 (EG1), *Experimental group 2 (EG2).
