# Supplementary material for: Cancer awareness among adolescents in second-level education: a mixed methods systematic review
Source: Health Educ Res. 2025 May 12;40(3):cyaf014. doi: 10.1093/her/cyaf014 (PMC12068056; doi:10.1093/her/cyaf014)
Supplement: cyaf014_Supp [file cyaf014_Supp.zip › Supplementary File SI D Quality appraisal.docx]

**Supplementary File Table SI D Quality** appraisal for studies included using the Mixed Methods Appraisal Tool (n=20)

| Category | Author & year | S1. Are there clear research questions? | S2. Do the collected data allow to address the research questions? | 1.1. Is the qualitative approach appropriate to answer the research question? | 1.2. Are the qualitative data collection methods adequate to address the research question? | 1.3. Are the findings adequately derived from the data? | 1.4. Is the interpretation of results sufficiently substantiated by data? | 1.5. Is there coherence between qualitative data sources, collection, analysis and interpretation? |
| --- | --- | --- | --- | --- | --- | --- | --- | --- |
| Qualitative descriptive | Woodgate & Busolo (2017) | Y | Y | Y | Y | Y | Y | Y |
| Category | Author | S1. Are there clear research questions? | S2. Do the collected data allow to address the research questions? | 2.1. Is randomization appropriately performed? | 2.2. Are the groups comparable at baseline? | 2.3. Are there complete outcome data? | 2.4. Are outcome assessors blinded to the intervention provided? | 2.5 Did the participants adhere to the assigned intervention? |
| Randomized Controlled Trial | Hubbard et al. (2016) | Y | Y | Y | Y | Y | Y | Y |
|  | Adamowicz et al. (2015) | Y | Y | Y | Y | Y | CT | Y |
|  | Stölzel et al. (2014) | Y | Y | Y | Y | Y | CT | Y |
|  | Lana et al. (2014) | Y | Y | Y | Y | Y | CT | Y |
| Category | Author | S1. Are there clear research questions? | S2. Do the collected data allow to address the research questions? | 3.1. Are the participants representative of the target population? | 3.2. Are measurements appropriate regarding both the outcome and intervention (or exposure)? | 3.3. Are there complete outcome data? | 3.4. Are the confounders accounted for in the design and analysis? | 3.5. During the study period, is the intervention administered (or exposure occurred) as intended? |
|  | Hudson-Rose et al.  (2023) | Y | Y | Y | Y | Y | Y | Y |
|  | Moskal et al.  (2023) | Y | Y | Y | Y | Y | Y | Y |
|  | Kerschner et al. (2022) | Y | Y | Y | Y | Y | Y | Y |
| Non-Randomized | Hudson et al. (2020a) | Y | Y | CT | Y | Y | CT | Y |
|  | Hudson et al. (2020b) | Y | Y | CT | Y | Y | CT | Y |
|  | Russell et al. (2020) | Y | Y | Y | Y | Y | Y | Y |
|  | Calderón- Garcidueñas et al. (2015) | Y | Y | CT | Y | Y | CT | Y |
|  | Kyle et al. (2013) | Y | Y | Y | Y | Y | N | Y |
|  | Kyle et al. (2013) | Y | Y | Y | Y | Y | N | Y |
| Category | Author | S1. Are there clear research questions? | S2. Do the collected data allow to address the research questions? | 4.1. Is the sampling strategy relevant to address the research question? | 4.2. Is the sample representative of the target population? | 4.3. Are the measurements appropriate? | 4.4. Is the risk of nonresponse bias low? | 4.5. Is the statistical analysis appropriate to answer the research question? |
| Quantitative descriptive | Abraham et al. (2021) | Y | Y | Y | Y | Y | Y | Y |
|  | Yildirim Usta and Ateskan (2020) | Y | Y | Y | Y | Y | Y | Y |
|  | Sugisaki et al. (2019) | Y | Y | Y | Y | Y | Y | Y |
|  | Al-Azri et al. (2019) | Y | Y | Y | Y | Y | Y | Y |
|  | Heuckmann et al. (2014) | Y | Y | Y | Y | Y | Y | Y |
|  | Kyle et al. (2012) | Y | Y | Y | CT | Y | CT | Y |

Abbreviations: CT=can’t tell; N=no; Y=yes.
